# Quantifying parent engagement in the randomized *Fuel for Fun* impact study identified design considerations and BMI relationships

**DOI:** 10.1186/s12874-021-01398-4

**Published:** 2021-10-09

**Authors:** Barbara Lohse, Leslie Cunningham-Sabo

**Affiliations:** 1 Rochester Institute of Technology, Wegmans School of Health and Nutrition, 180 Lomb Memorial Drive 78-A622, Rochester, NY 14623 USA; 2 Department of Food Science and Human Nutrition, Colorado State University, 106 Gifford Building, Fort Collins, CO 80523-1571 USA

**Keywords:** Parent engagement, School-age youth, Childhood obesity prevention, Nutrition education, Index development

## Abstract

**Background:**

Parent participation in children’s health interventions is insufficiently defined and measured. This project quantified parent participation to enable future examination with outcomes in an intervention focused on 4th graders, aged 9–11 years, and their families living in northern Colorado.

**Methods:**

Indices were developed to measure type (Parent Participation Profile; PPP) and intensity (Parent Engagement Intensity; PEI) of engagement in *Fuel for Fun (FFF)*, an asymmetric school-and family-based intervention for 4th graders. Study arm-specific participation opportunities were catalogued and summed to calculate the PPP. An algorithm considered frequency, effort, convenience, and invasiveness of each activity to calculate PEI. Indices were standardized (0–100%) using study arm-specific divisors to address asymmetric engagement opportunities. Parents who completed ≥75% of the PPP were defined as Positive Deviants. Youth height and weight were measured. Youth BMI percentile change was compared with parent Positive Deviant status using general linear modeling with repeated measures that included the participation indices.

**Results:**

Of 1435 youth, 777 (54%) had parent participation in at least one activity. Standardized means were 41.5 ± 25.4% for PPP and 27.6 ± 20.9% for PEI. Demographics, behaviors or baseline *FFF* outcomes did not differ between the Positive Deviant parent (n = 105) and non-Positive Deviant parents (n = 672); but more Positive Deviant parents followed an indulgent feeding style (*p* = 0.015). Standardized intensity was greater for Positive Deviant parents; 66.9 ± 20.6% vs 21.5 ± 12.7% (*p* < 0.001) and differences with non-Positive Deviant parents were related to activity type (*p* ≤0.01 for six of eight activities). Standardized participation intensity was associated with engagement in a greater number of standardized activity types. Among participating parents, standardized intensity and breadth of activity were inversely related to the youth BMI percentile (n = 739; PEI r = −0.39, *p* < 0.001; PPP r = −0.34, *p* < 0.001). Parent engagement was not associated with parent BMI change.

**Conclusions:**

An activity-specific intensity schema operationalized measurement of parent engagement in a complex, unbalanced research design and can serve as a template for more sensitive assessment of parent engagement. Positive deviance in parent engagement was not a function of personal, but rather activity characteristics. PPP and PEI increased with fewer requirements and convenient, novel, and personalized activities. Parent engagement indices affirmed lower engagement by parents of overweight/obese youth and concerns about target reach.

## Background

Programs and interventions that address nutrition, obesity prevention, physical activity and other health-related issues for school-age youth recognize the value of parent engagement in tandem with youth involvement [1]. Parent participation features are considered in program development and methods to assure, motivate, personalize, and diversify parent engagement have been rigorously investigated [2–11]. Interviews and focus groups with target parents to learn of incentivizing factors may precede program development or implementation to better court parent participation [8, 12–15]. However, the current consensus is that parent engagement is understudied, marginally utilized, and challenging to define and measure [4, 7, 16, 17]. In addition, parent engagement has been described as a “dynamic construct that changes over time in complex ways,” ([18] p. 811) influenced by neighborhoods and organization climate [14] with different strategies applicable to recruitment, enrollment, retention, and participation stages of a program [19].

These challenges are reflected in the way parent engagement is included in assessing program outcomes. For example, attendance or intention to participate dominate assessments of parent involvement followed by socio-economic and demographic descriptors of participating parents with few to no reports of participation quality or intensity [4, 5, 7, 9–11, 17, 19]. In a literature review of 24 studies of parent engagement in child and family mental health treatments, 17 included at least one measure of engagement; the maximum number of measures was three. These studies reported participation in general or with specific measures e.g., resistance, enthusiasm, collaboration with a provider or verbalization and also completion of tasks to be completed at home [7]. Studies specifically targeting obesity prevention for school-age youth include parent engagement in their program descriptions, but have ignored or minimized inclusion in outcome analyses [7, 20–23]. *Fuel for Fun (FFF*) was a multi-component program for 4th grade youth that included cooking skills, physical activity, and food culture to address obesity prevention tenets. *FFF* was delivered over one academic year to youth in 8 schools in 4 cohorts with no treatment in cohorts 1 and 4 and *FFF* in cohorts 2 and 3. Program impact was examined in a cluster-randomized study design with assessments at baseline, post-program and 1 year following baseline. Primary student outcomes were fruit and vegetable preference, self-efficacy for preparing food, and attitude toward cooking and eating fruits and vegetables. Four school- and cohort-specific parent treatment arms provided asymmetric participation options (described in Table 1) and an opportunity to include parent engagement in youth outcome assessment [24].

**Table 1:** Values to Calculate Cohort- and School-based Parent Participation Profiles and Parent Engagement Indices

School	Possible Activity Types	Maximum PPP^a^			Maximum PEI^b^		
		Total	Prog	Eval	Total	Prog	Eval
Cohort 1^c^							
1	Surveys Accelerometer	2	0	2	12	0	12
2	Surveys	1	0	1	6	0	6
3	Surveys Accelerometer	2	0	2	12	0	12
4	Surveys *About Eating*	2	1	1	12	6	6
5	Surveys *About Eating*	2	1	1	12	6	6
6	Surveys *About Eating*	2	1	1	12	6	6
7	Surveys	1	0	1	6	0	6
8	Surveys Accelerometer *About Eating*	3	1	2	18	6	12
Cohort 2^c^							
1	Surveys Parent Diet Assessment Youth Diet Assessment Accelerometer Recipe Use	5	1	4	30	6	24
2	Surveys Parent Diet Assessment Youth Diet Assessment Family Night Action Pack Recipe Use	6	3	3	36	18	18
3	Surveys Parent Diet Assessment Youth Diet Assessment Accelerometer Recipe Use	5	1	4	30	6	24
4	Surveys Parent Diet Assessment Youth Diet Assessment Family night Action Pack *About Eating* Recipe Use	7	4	3	42	24	18
5	Surveys Parent Diet Assessment Youth Diet Assessment *About Eating* Recipe Use	5	2	3	30	12	18
6	Surveys Parent Diet Assessment Youth Diet Assessment *About Eating* Recipe Use	5	2	3	30	12	18
7	Surveys Parent Diet Assessment Youth Diet Assessment Family Night Action Pack Recipe Use	6	3	3	36	18	18
8	Surveys Parent Diet Assessment Youth Diet Assessment Accelerometer Family Night Action Pack *About Eating* Recipe Use	8	4	4	48	24	24
Cohort 3^c^							
1	Surveys Parent Diet Assessment Youth Diet Assessment Accelerometer Recipe Use	5	1	4	30	6	24
2	Surveys Parent Diet Assessment Youth Diet Assessment Family Night Action Pack Recipe Use	6	3	3	36	18	18
3	Surveys Parent Diet Assessment Youth Diet Assessment Accelerometer Recipe Use	5	1	4	30	6	24
4	Surveys Parent Diet Assessment Youth Diet Assessment	3	0	3	18	0	18
5	Surveys Parent Diet Assessment Youth Diet Assessment *About Eating* Recipe Use	5	2	3	30	12	18
6	Surveys Parent Diet Assessment Youth Diet Assessment *About Eating* Recipe Use	5	2	3	30	12	18
7	Surveys Parent Diet Assessment Youth Diet Assessment Family Night Action Pack Recipe Use	6	3	3	36	18	18
8	Surveys Parent Diet Assessment Youth Diet Assessment Accelerometer Family Night Action Pack *About Eating* Recipe Use	8	4	4	48	24	24
Cohort 4^c^							
1	Surveys Accelerometer Parent Diet Assessment Youth Diet Assessment	4	0	4	24	0	24
2	Surveys Parent Diet Assessment Youth Diet Assessment	3	0	3	18	0	18
3	Surveys Accelerometer Parent Diet Assessment Youth Diet Assessment	4	0	4	24	0	24
4	Surveys Parent Diet Assessment Youth Diet Assessment	3	0	3	18	0	18
6	Surveys *About Eating* Parent Diet Assessment Youth Diet Assessment	4	1	3	24	6	18
7	Surveys Parent Diet Assessment Youth Diet Assessment	3	0	3	18	0	18
8	Surveys Accelerometer *About Eating* Parent Diet Assessment Youth Diet Assessment	5	1	4	30	6	24

*PPP* Parent Participation Profile, *PEI* Parent Engagement Index, *Prog Program*, *Eval* Evaluation

^a^ Divisors for standardized PPP calculation

^b^ Divisors for standardized PEI calculation. Is the sum of PEI for each activity type

^c^ Cohorts 1 and 4 were controls; Cohorts 2 and 3 were Fuel for Fun intervention

Activity type PEI is product of frequency x intensity factor. See Table 2 for frequency and intensity factor details, Table 3 for sample calculations

### Study purpose

The purpose of this study was to critically examine *FFF* parent engagement options to quantify their engagement, which will then facilitate careful consideration of parent engagement influences on *FFF* impact and to serve as a model for evaluation of parent engagement in other youth-based programs.

## Methods

### Research design

This descriptive study catalogued and tabulated parent engagement actions from parents of 4th grade youth, aged 9–11 years, living in northern Colorado participating in a cluster-randomized impact assessment of *Fuel for Fun* (*FFF*) [24] during 2013–2018.

The FFF curriculum, based on concepts from Social Learning Theory and Experiential Learning Theory, includes five cooking and five tasting lessons, a focus on physical activity at recess, and promotions in the school lunch program. In addition, four schools participated in a family component that included two evening events at the school and home-based activities to be completed by parents with their child. FFF impact was studied by comparing four year-long cohorts with years 1 and 4 acting as controls and years 2 and 3 receiving the FFF intervention. Two schools in years 2 and 3 had an added family component to the FFF program, three schools participated in an accelerometry activity during control and FFF years and three cohorts had the opportunity to participate in dietary assessments. Thus, parents had varying levels of possible engagement, which were unbalanced among years and even between FFF participants.

### Data collection

Event activity logs and online evidence of parent participation were examined. Logs were from accelerometer use, family fun night attendance, completion of take-home action packs that continued classroom activities, self-report of classroom-based recipe use and youth telephone-based diet recalls that needed parent assistance to complete. Online evidence included the ASA24 parent dietary record, accessing *About Eating,* an online nutrition education program [25] and completion of an online survey set that included nine validated survey instruments [24] in addition to self-reported weight and height and psycho-sociodemographic questions. Youth weight and height were measured by trained research staff using a digital scale to the nearest tenth of a kilogram and a portable stadiometer to the nearest tenth of a centimeter. Informed consent was obtained from all parents that accessed the online survey and dietary assessment platforms. The survey captured action pack completion and family fun night attendance, but some parents attended family fun nights and/or completed action packs without accessing the online consent form that prefaced the survey. These two activities were part of the educational curriculum of the school and parents signed an attendance/completion record that enabled us to count their participation. Parents completed a separate consent form for their child’s study participation that included consent for accelerometry for themselves and their child. All students, whose parents consented to their child’s participation also signed an assent form prior to collection of any student information.

Measures were obtained at baseline at the start of the 4th grade school year, seven months later in spring, which was immediately following completion of the intervention for the *FFF* cohorts, and in the fall of the next (5th grade) school year.

All study methods were carried out in accordance with relevant guidelines and regulations. Data collection procedures were reviewed and approved [IRB # 19-9204H] by the Colorado State University Institutional Review Board for the Protection of Human Subjects and the Rochester Institute of Technology Institutional Review Board for the Protection of Human Subjects. Informed consent was obtained in writing from the parents. Parents completed an informed consent for their child’s participation and the students also signed an assent form prior to collection of any student information.

### Development of parent engagement measures

Two parent engagement indices were calculated: Parent Participation Profile (PPP) and Parent Engagement Intensity (PEI). The PPP was a *sum* of the *number* of *types* of activities a parent attempted, which are listed in Table 2. Since some parents had an opportunity to engage in more *types* of activities, which was school and cohort dependent, the PPP was standardized by dividing it by the number of activities offered each parent and ranged from 0 to 100%. The PEI characterized engagement by indicating *intensity* of *involvement*, based on frequency and burden of involvement for each of the eight activities with a possible range of 2–48*.* Owing to school and cohort-based differences in engagement opportunities, the PEI, like the PPP, was standardized by dividing by the possible level of intensity. Burden of involvement was represented by an intensity factor that considered five effort-related issues: 1) incentive involved? 2) being away from home required? 3) engagement with the child required? 4) ≥ 30 min required? and 5) personal information needed? For example, family fun nights had an intensity factor of 3 because they required being away from home (+1), engaging with the child (+1) and lasting ≥ 30 min (+1). Possible frequency of involvement was based on actual occurrence (e.g., 3 surveys, or logging in to view 6 *About Eating* lessons) or, for items with multiple opportunities a scale based on the perceived amount of required attention required (e.g., prepared 1 recipe- 1 point; prepared 2 or 3 recipes-2 points; prepared 4 or the maximum 5, recipes-3 points). A shown in Table 2, the weighting, i.e., the product of the frequency and the intensity factor, was identical for each of the 8 items. School and cohort-based PPP and PEI calculation details are shown in Table 1 with sample calculations in Table 3.

**Table 2:** Parent Engagement Profile Activities and Factors Contributing to Parent Engagement Intensity

Activity Type	Possible Frequency	Intensity Factor ^a^					Weighting ^b^
		Incentive?	Away from Home?/Internet?	Involves Child?	Estimated Time	Personal Information	
Program Activities							
Family Fun Nights	2 events	Y = 0	Y = 1	Y = 1	2.0 h = 1	N = 0	2 X 3 = 6
Action Packs ^c^	10 packs	N = 1	N = 0	Y = 1	0.17 h = 0	N = 0	3 X 2 = 6
Recipe Preparation ^d^	5 recipes	Y = 0	N = 0	Y = 1	0.50 h = 1	N = 0	3 X 2 = 6
*About Eating*	6 modules	N = 1	N = 0	N = 0	0.25 h = 0	N = 0	6 X 1 = 6
Evaluation Activities							
Parent Survey	3 time points	Y = 0	N = 0	N = 0	0.50 h = 1	Y = 1	3 X 2 = 6
Parent Diet Assessment ^e^	3 recalls X 3	Y = 0	N = 0	N = 0	0.25 h = 0	Y = 1	6 X 1 = 6
Youth Diet Assessment ^e^	3 recalls X 3	Y = 0	N = 0	Y = 1	0.25 h = 0	N = 0	6 X 1 = 6
Accelerometry	3 time points	Y = 0	N = 0	N = 0	1.00 h = 1	Y = 1	3 X 2 = 6

^a^ Rationale for defining intensity; assign 0 is parent paid, 1 if not paid; 0 if done at home/on internet, 1 if done away; 0 if didn’t involve child, 1 if involved child; 1 if >30 (0.5 h) minutes to complete, 0 if less; and 1 if personal information requested and 0 if not

^b^ Weighting = Frequency X Sum of intensity values

^c^ Take home activity packs that aligned with classroom lessons/activities to be completed by parent with child and signed completion verification returned to school; 1 to 3 action packs = 1 point; 4–6 action packs = 2 points; 7–10 action packs = 3 points

^d^ 1 recipe =1 point; 2 to 3 recipes =2 points; 4 to 5 recipes =3 points

^e^ For each of 3 possible time points 1 recall =1 point, 2 or 3 recalls =2 points

**Table 3:** Sample Calculations of Maximal Standardized Parent Participation Profiles and Parent Engagement Intensity with Examples of Participation

	Possible Activities (Possible Frequencies)	Maximal PPP Standardized PPP^a^	Maximal PEI Standardized PEI^b^
School A control cohort	Parent Surveys (3) **Example – parent completed** **2 surveys**	1 **1/1 = 100%**	6 **(2 + 2)/6 = 66%**
School B control cohort	*About Eating* lessons (6) Parent Surveys (3) **Example – parent completed 4** ***About Eating*** **lessons and 1 survey**	2 **2/2 = 100%**	12 **(4 + 2)/12 = 50%**
School C *FFF* cohort	*About Eating* lessons (6) Recipes (5) Parent Surveys (3) Parent Diet Assessment (3) Youth Diet Assessment (3) **Example – parent completed 3** **surveys, 3 youth diet assessments (2 recalls each), and 1** ***About Eating*** **lesson**	5 **3/5 = 60%**	30 **(2 + 2 + 2 + 2 + 2 + 2 + 1)/30 = 43.3%**
D – *FFF* year	Family Nights (2); Action Pack (10) Recipes (5); *About Eating* lessons (6) Parent Surveys (3); Accelerometer (3) Parent Diet Assessment (3) Child Diet Assessment (3) **Example – Parent completed 5 action packs,** **4** ***About Eating*** **lessons, 2 parent surveys,** **2 parent diet assessment (2 recalls each) and 2 youth diet assessments (3 recalls each)**	8 **5/8 = 62.5%**	48 **(2 + 4 + 2 + 2 + 2 + 2 + 2 + 2)/48 = 37.5%**

*PPP* Parent Participation Profile, *PEI* Parent Engagement Intensity

^a^ Count activity types to calculate PPP; Divide parent PPP by maximal PPP possible to calculate standardized PPP

^b^ Multiply Frequency (in parentheses) by the Intensity Factor to calculate PEI; Divide PEI by maximal PEI possible to calculate standardized PEI

Frequency: 1 to 3 action packs = 1 point; 4–6 action packs = 2 points; 7–10 action packs = 3 points; 1 recipe =1 point; 2 to 3 recipes =2 points; 4 to 5 recipes =3 points; At each of 3 time points 1 diet recall =1 point, 2 or 3 diet recalls =2 points

Intensity Factor: Family Nights 3; Action Packs 2; Recipe Preparation 2; *About Eating* 1; Parent Survey 2; Parent Diet Assessment 1; Youth Diet Assessment 1; Accelerometry 2

Four of the eight possible activities were program components and four were evaluation tasks (Table 2). PPP, PEI, and their standardized formats were divided into programmatic and evaluation contributions and compared using Pearson correlation coefficients to ascertain if intensity of involvement was related to breadth of engagement.

A relatively new concept to frame understanding of health problems is *Positive Deviance* (PD)*.* Applied to health practice, PD has been characterized as the presence of highly desired performance in a behavioral domain, which can then be studied for translation to problem solving strategies [26]. Parent engagement rates across studies and target audiences vary considerably, but two programs declared as “high,” their engagement rates of 74% [11] and 80% [14]. Thus, based on these studies and prior experience with *FFF,* a standardized PPP or PEI ≥ 75% were defined as PD in parent engagement.

Scoring guidelines were followed for each scale in the parent survey set, including the International Physical Activity Questionnaire [27], Child Feeding Styles Questionnaire [28], Parent Perceived Stress [29], Eating Competence [30, 31], Fruit and Vegetable Availability [32], Modeling Healthful Eating [33] and Self-Efficacy/Outcome Expectancies for serving fruits and vegetables [34]. Healthy Eating Indices were calculated from parent and student 24-h dietary recalls using the 2010 Dietary Guidelines [35, 36].

Independent t-tests, ANOVA and Chi-square were used to compare participating non-PD and PD parents and to compare students who had parents with any level of engagement to those whose parents did not participate at all. Psycho-demographic and *FFF* treatment group differences were tested with Independent t-tests and ANOVA; correlations were assessed with the Pearson correlation coefficient. Since youth BMI percentile differed among the eight schools (*p* = 0.001) and 38 classrooms (*p* = 0.023), youth BMI percentile was adjusted for school and classroom enrollment. Change in BMI percentile over 12 months was examined using GLM repeated measures comparing PD with non-PD parents and reporting group marginal means and standard errors.

## Results

### Description of participants and engagement

Of the 1435 consented youth, 7 did not provide a survey, but a parent was in the study. The resulting sample of fourth grade youth participants (n = 1428, 711 males) were mostly white (n = 1065, 75%) with 17% (n = 238) Hispanic, 5% (n = 66) reporting 2 or more races, and ≤ 2% each for American Indian, Asian, Black, and Hawaiian/Pacific Islander. Mean age was 9.6 ± 0.4 y and mean BMI percentile was 56.6 ± 30.1 with 24% (n = 335) above the 85th percentile and 11% (n = 148) above the 95th percentile.

Demographic information was not available for parents who participated only in activities other than the survey (e.g., accelerometry, family nights) because the survey was the source of demographic and behavioral information. As shown in Table 4, parents were highly educated and reported being physically active. Of the 413 parents completing the survey, mean age was 39.1± 5.9y, (range 25 – 65y, n = 411), 86% of the 410 reporting gender were female and 50% noted their 4th grader was a girl. Parents were mostly white (94%) with 8% Hispanic (n = 32; 1 Hispanic Black/31 Hispanic White). Fewer than 5% were Asian, American Indian, Hawaiian/Pacific Islander or Black. Additional psychosocial characteristics are shown in Table 4.

**Table 4:** Characteristics of Parent Survey Completers (n = 413) Described and Compared by Positive Deviance Status as Measured by the Standardized Parent Participation Profile

Baseline Psychosocial Factors	Standardized Parent Participation Profile^a,b^		
	Total group	PD (n = 105)^c^	Non-PD (n = 308)
ecSI 2.0™ ^d^ (n = 397)	32.2± 8.1	33.2 ± 7.9 (n = 101)	31.8±8.1
Subscales:			
Eating Attitudes^e^	12.9±3.5 (n = 406)	13.1±3.4 (n = 102)	12.8±3.6
Food Acceptance ^f^*	5.1±2.0 (n = 407)	5.5±2.1 (n = 101)	5.0±2.0
Internal Regulation^g^	4.0±1.4 (n = 410)	4.0±1.5 (n = 102)	4.0±1.4
Conceptual Skills^h^	10.2±3.0 (n = 409)	10.6±2.7 (n = 102)	10.1±3.0
EC ^i^ (n = 397)	210 (53%)	58 (57%)	152 (51%)
BMI ^j^ (n = 407)	26.1±5.8	25.5±5.5 (n = 102)	26.3±6.0
Underweight (n = 6)	6 (1%)	2 (2%)	4 (1%)
Normal weight (n = 207)	207 (51%)	55 (54%)	152 (50%)
Overweight (n = 117)	117 (29%)	27 (26%)	90 (30%)
Obese (n = 77)	77 (19%)	18 (18%)	59 (19%)
Highest Education			
High School or less	25 (6%)	7 (7%)	18 (6%)
Some College/Training	119 (29%)	26 (25%)	93 (30%)
4-yr College Degree	145 (35%)	40 (38%)	105 (34%)
Post graduate College	124 (30%)	29 (28%)	95 (31%)
Used ≥1 Assistance Program	120 (29%)	32 (30%)	88 (29%)
Child Feeding Style^k^ **			
Uninvolved	78 (19%)	12 (12%)	66 (22%)
Indulgent	120 (30%)	41 (42%)	79 (26%)
Authoritative	83 (21%)	17 (17%)	66 (22%)
Authoritarian	120 (30%)	28 (29%)	92 (30%)
Physical Activity Level^l^			
Low	94 (23%)	22 (21%)	72 (23%)
Moderate	119 (29%)	28 (27%)	91 (30%)
High	200 (48%)	52 (50%)	148 (48%)
Modeling Score^m^ (n = 403)	15.2±4.2	15.7±3.5 (n = 102)	15.0±4.4(n = 301)
SE/OE Score^n^ (n = 405)	53.1±8.8	53.6±9.0(n = 100)	52.9±8.7(n = 305)
HEI score^o^ (n = 77)	55.4±12.4	55.2±12.1(n = 54)	56.0±13.6(n = 23)
Stress^p^ (n = 426)	6.7±2.1	6.7 ± 2.0	6.7±2.1 (n = 321)

Table entries are mean (Standard Deviation) or frequencies (%). Percentages may not sum to 100% because of rounding. Sample sizes are as listed in the heading unless included in parentheses EC Eating Competent, SE/OE Self-efficacy/Outcome Expectancies toward fruits and vegetables, ecSI 2.0™ Satter Eating Competence Inventory 2.0,™ PD Positive Deviant

^a^ Calculated by dividing Parent Participation Profile (PPP) by number of activities offered

^b^ Column percentages are proportion of total sample or sample sizes specific to each psychosocial factor of Positive Deviant and non-Positive Deviant parents respectively

^c^ Positive deviant parent defined as participating in 75% or more of available activity types

^d^ Satter Eating Competence Inventory 2.0™ possible score 0–48; higher scores indicate more eating competence

^e^ Eating Attitudes subscale possible range 0–18

^f^ Food Acceptance subscale possible range 0–9

^g^ Internal Regulation subscale possible range 0–6

^h^ Contextual Skills subscale possible range 0–15

^i^ Eating Competence defined as ecSI 2.0™ ≥ 32

^j^ BMI calculated from self-reported weight and height

^k^Assessed by Child Feeding Styles Questionnaire; Total n = 401; positive deviant n = 98

^l^ Measured with the International Physical Activity Questionnaire

^m^ Possible score 0–33; higher scores indicate more modeling

^n^ Possible score 12–60; higher scores indicate greater SE/OE

^o^ Healthy Eating Index, Calculated using 2010 Dietary Guidelines

^p^ Possible score 1 (no stress) to 10 (extreme stress)

^*^ Independent t-test comparing Positive Deviant status *p* = 0.024

^**^Chi Square comparing Positive Deviant status *p*=0.015

Eight activity types were identified. Of the 1435 students, 777 (54%) had a parent who participated in at least 1 activity with a mean of 1.9 ± 1.2 types of activity (range 1–7). These parents participated in nearly half of the activity types available to them; mean standardized PPP was 41.5% ± 25.4% (range 13–100%). The mean intensity of participation or PEI, 7.5 ±5.7 (range 2–30), was slightly more than one-fourth (27.6% ±20.9%; range 4–100%) of the possible intensity level (i.e., standardized PEI). Overall, those who did more of the available activity types engaged in them at a higher intensity (r = .87, *p* < 0.001, n = 777). Of engaged parents, 105 (14%) did 75% or more (defined as PD) and 76 (10%) did every activity they were offered. However, only 39 (5%) participated at 75% or more of the possible intensity and 16 (2%) at their highest possible intensity. Participation in evaluation activity options was greater and more intense than in the program activities (46.0 ± 30.7% vs 24.5 ± 30.8%; 30.9 ± 24.7% vs 12.6 ± 19.0%) but breadth and intensity of evaluation and program activities were significantly related (PPP r = 0.18, *p* < 0.001; PEI r = .27, *p* < 0.001).

Students were evenly distributed between control (n = 740; 52%) and *FFF* (n = 695; 48%), but participating parents, compared to non-participants, had youth in the *FFF* treatment arm (57% vs 38%; *p* < 0.001). Compared to controls, parents with youth in *FFF* engaged in more activity types (PPP 2.2 ± 1.4 vs 1.6 ±0.9; *p* < 0.001) with greater intensity (PEI 8.2 ± 6.1 vs 6.6± 4.8; *p* < 0.001). However standardized scores showed that *FFF* parents did only 32.9% ± 19.6% of possible activity types compared to 53.0% ± 27.6% for controls (*p* < 0.001). In addition the standardized intensity level was lower (*p* < 0.001) in parents with youth in the *FFF* treatment (20.9% ± 15.1% vs. 36.7% ± 24.0%;*p* < 0.001).

### Parent engagement and activity types

Participating parents doing <25% of what was available to them (n = 128) engaged in only four of the eight activity types and these four were the same activities that involved more than 15 participants for the 285 parents who engaged in 25–49.9% of the available activities (Table 5). Compared to less engaged parents, PD had a significantly greater proportion of participation in all activities except for accelerometry and take-home action packs (Table 6). Both of these activities did not require survey completion to engage and were the two most common activities notwithstanding the online parent survey. Dietary assessment for both youth and parents was popular; both were incentivized and not time intensive. PD participation in *About Eating* and Recipe Prep was similar even though the online program was not incentivized and had many modules, whereas recipe preparation was incentivized by providing a key ingredient, involved the child and took more than 30 min to do.

**Table 5:** Activity Type Participation Compared Among Categories of Standardized Parent Participation Profiles

	n	Standardized Parent Participation Profile^a^			
		0–24.9% n = 220	25–49.9% n = 285	50–74.9% n = 167	75–100% ^b^ n = 105
Program Activities					
Family Fun Nights	95	30	26	26	13
Action Packs^c^	185	37	89	43	16
Recipe Preparation	53	0	9	27	17
*About Eating*	70	0	15	22	33
Evaluation Activities					
Parent Survey	432	38	158	131	105
Parent Diet Assessment	79	0	4	18	57
Youth Diet Assessment	101	0	7	35	59
Parent Accelerometry	459	115	181	106	57

Table entries are frequencies

^a^ Calculated by dividing Parent Participation Profile (PPP) by number of activities offered

^b^ Positive deviant parent (75–100% of activities offered)

^c^ Take home activity packs that aligned with classroom lessons/activities to be completed by parent with child and a signed completion verification was returned to school

**Table 6:** Activity Participation Compared by Positive Deviance Status as Measured by the Standardized Parent Participation Profile

	Standardized Parent Participation Profile^a^	
	PD (n = 105) ^b,c^	Non-PD (n = 672)
Program Activities		
Family Fun Nights (n = 95)	13 (12%)	82 (12%)
Recipe Preparation (n = 53)^**^	36 (34%)	17 (3%)
Action Packs^d^ (n = 185)^*^	16 (15%)	169 (25%)
*About Eating* (n = 70)^**^	33 (31%)	37 (5%)
Evaluation Activities		
Parent Survey (n = 432)^**^	105 (100%)	327 (49%)
Parent Diet Assessment (n = 79)^**^	57 (54%)	22 (3%)
Child Diet Assessment (n = 101)^**^	59 (56%)	42 (6%)
Accelerometry (n = 459)	57 (54%)	402 (60%)

Table entries are frequencies (%)

^a^ Calculated by dividing (PPP) by number of activities offered

^b^ PD Positive deviant parent defined as participating in 75% or more of available activity types

^c^ Column percentages are proportion of PD or non-PD parents participating in the listed activity

^d^ Take home activity packs that aligned with classroom lessons/activities to be completed by parent with child and signed completion verification returned to school.^*^ Chi Square between activity participation or not and Positive Deviant status *p* <0.05

^**^ Chi Square between activity participation or not and Positive Deviant status *p* < 0.001

PD standardized PEI was higher than non-PD (66.9 ± 20.6% vs. 21.5 ± 12.7%; *p* < 0.001) with a significantly greater PD PEI for the four evaluation activities and two of the program activities: Parent (2.2 ± 2.4 vs 0.1 ± 0.8; *p* < 0.001) and child (2.8 ± 2.7 vs 0.2 ± 1.0; *p* < 0.001) dietary assessments, accelerometry (4.4 ± 1.6 vs. 3.8 ± 1.9; *p* = .01), online survey completion (4.7 ± 1.6 vs. 4.0 ± 1.7; *p* < 0.001), *About Eating* (0.9 ± 1.8 vs 0.1 ± 0.7; *p* < 0.001), and recipe preparation (0.5 ± 1.3 vs .02 ± 0.8; *p* < 0.001).

More youth in the *FFF* group had participating parents than control youth (64% vs 45%; *p* < .001). The only differences between PD and non-PD parents was that PD parents had fewer children in the *FFF* group than non-PD parents (27% vs 50%; *p* < 0.001), and as shown in Table 4, their Food Acceptance subscale score of the Satter eating competence inventory was higher and child feeding styles were different. Compared to non-PD parents, PD parents were indulgent and less uninvolved. As shown in Table 6, PD more frequently than non-PD participated in parent and youth dietary assessments, recipe use and the online survey. Fewer PD than non-PD parents completed Action Packs but among the Action Pack participants, the difference in number completed (which could range from 1 to 10) was not significant between PD and non-PD parents (3.1 ± 2.6 vs 3.5 ± 2.6; *p* = .50). No other differences were noted in demographics, stress level, eating competence, activity level, or other eating behaviors (Table 4).

### Relationship between BMI and parent engagement

Obese/overweight parent(n = 198) PPP, PEI, and standardized values did not differ from parents with normal/underweight (n = 228) BMI. BMI percentile of youth with engaged parents (n = 737) was significantly lower than youth without participating parents (n = 636; 55.5 ± 8.0 vs. 58.1 ± 7.0; *p* < 0.001). Youth BMI percentile was lower for those whose parents participated in all the available activities (n = 74) compared to those with 1–99% engagement (49.2 ± 10.0 vs. 56.2 ± 8.3; *p* < 0.001). Of the youth with engaged parents, 174 (23%) were overweight or obese at baseline; their parent PEI and PPP were lower than parents of normal/underweight youth (6.5 ± 5.0 vs 7.8 ± 5.9, *p* = 0.005, 1.7 ± 1.1 vs 2.0 ± 1.3, *p* = 0.019).

Of all youth, 354 (26%) were overweight or obese at baseline. Pattern of change in adjusted BMI percentile over 12 months did not differ between youth of PD and non-PD parents. However, BMI percentile for youth of PD (n = 85) was lower (*p* < 0.001) at baseline (49.0 ±0.8) and 12 months later (48.7± 0.8) than for non-PD parents (n = 1015; 57.1 ± 0.2, 56.9 ± 0.2 respectively). Among participating parents, standardized intensity and breadth of activity were inversely related to the youth BMI percentile, (n = 739; PEI r = −.0.39, *p* < 0.001; PPP r = −0.34, *p* < 0.001).

## Discussion

Parent engagement metrics of 1435 youth were examined by weighting the burden of each activity type to extend the description of parent engagement from participation type (PPP) to an intensity-driven value (PEI). PPP and PEI were standardized to adjust for the asymmetric opportunities for engagement. At 54%, parent participation was at a higher rate than others [6, 14], possibly reflecting activity attributes that have been suggested by others to enhance engagement, e.g., parent perceptions of relevance and usefulness, targeted cooperation between home and school and a focus on parent needs [13]. Recipe use was high for engaged parents, congruent with findings from Burrows et al. [2] which also aligns with interest of involving the child and doing something useful and relevant to a parent’s life. Online activities attract parents [2, 4], and indeed *About Eating*, an online option, was popular with PD parents, supporting the conclusion that innovative, personal, and accessible activities can enhance participation. The mosaic of activity burden demonstrated that it is not one barrier or issue that promoted or impeded engagement. However, activities with higher engagement levels shared features of being innovative or novel, easy to access and personally applicable. Unlike less engaged parents, PD extended the attraction to novel, accessible, and personal activities (e.g., accelerometry, online survey, recipe preparation) to ones with the same features that also required greater cognitive and emotional investment, e.g., dietary assessments, *About Eating*. The influential character of payment to incentivize participation is unclear [1, 5]. In this study the incentivized activities were more frequently utilized, but of note is that participation in the more incentivized evaluation activities was significantly related to participation in the less incentived programmatic options. In fact, participation in more activity types (i.e, > PPP) was strongly associated with being more intensely involved (i.e., > PEI).

In concurrence with Burrows et al. [2], socio-economic factors did not differ between PD and non-PD parents. However, others have shown that parent attendance is associated with being more educated and of a higher socioeconomic status [2, 7, 21], suggesting a need for further study.

The literature is dense with studies that describe parent engagement strategies, but practically devoid of examples where parent engagement was included in the analysis of primary research outcomes. A Cochrane Review of 55 trials (involving more than 11,000 people) designed to increase fruit and vegetable intake of youth five years old or younger concluded that parent nutrition education could not be linked to increasing youth fruit and vegetable consumption [37]. If parent engagement measures reported in the literature of school age youth are indicative of those used in these 55 studies with preschool age youth, then the Cochrane conclusion to include more rigor in future research should include directives to better quantify and describe parent engagement. Heredia et al., [8] reported that parent social support explained 9–13% of variance in children’s energy balance-related behaviors. Values this high can affect conclusions about program efficacy or performance and support efforts to quantify intensity of parent engagement. This call to action is soundly supported by the finding that youth BMI was associated with parent engagement, despite no such relationship with parent BMI. Being able to show that overweight or obese youth had less engaged parents (both in types of activities and intensity) can create an added dimension to intervention analytic strategy and inform program revision. The relationship between BMI and parent engagement was not shown in a systematic review of parent engagement in child obesity prevention interventions that suggested anthropometric indices as an inappropriate signal of parent engagement effectiveness [38]. However, the measures of parent engagement observed in the systematic review appeared to have limited calculation sensitivity and thus the requisite power to characterize parent engagement. The current study suggests that measurement of parent engagement may be instrumental for interpretation of outcomes from youth programs focused on child weight as well as for identifying successful reach.

### Study limitations and strengths

This study was limited by the research design that required survey completion for participation in some activities and not others (e.g. accelerometry, recipes). Thus, demographic and behavioral data to describe parents without a survey are not available. A small number of engaged parents did not have a youth participant owing to absence or refusal that precluded linking parent data with youth. Parent weight and height are self-reported; measured height and weight are recommended for future studies. Additionally, findings may not be generalizable to low-income, less educated, and physically inactive parents. However, this study was unique, i.e., the asymmetric opportunities for involvement facilitated examination of parent engagement strategies and the opportunity to participate in an activity occurred more than once allowing for intensity assessment. Other strengths included participation activities for one year with three critically spaced measurement episodes. In addition, youth heights and weights were objectively measured by trained personnel. Further study of methods to quantify parent engagement and to examine change in intensity over a longer period of time is recommended.

## Conclusions

Measuring intensity of engagement in addition to tabulating presence of participation is a valuable way to define or learn about level of involvement. For example, although parents participated in nearly half of the available activities, level of involvement was reconsidered because intensity of participation was only one-fourth of the possible level. Understanding the influence of parent engagement on program impact or health-related behaviors is a key motive for better defining and measuring it for inclusion in program evaluation. This project provided a novel approach to quantifying parent engagement that addressed intensity as well as breadth, which will increase in pertinence as research designs of intervention impact studies become more complex to accommodate limitations in time, funding, and sample characteristics.

## Data Availability

The datasets used and/or analyzed during the current study are available from the corresponding author on reasonable request.

## Abbreviations

FFF: Fuel for Fun
PPP: Parent Participation Profile
PEI: Parent Engagement Intensity
PD: Positive Deviance
